# Antibodies Targeting Novel Neutralizing Epitopes of Hepatitis C Virus Glycoprotein Preclude Genotype 2 Virus Infection

**DOI:** 10.1371/journal.pone.0138756

**Published:** 2015-09-25

**Authors:** Kai Deng, Ruyu Liu, Huiying Rao, Dong Jiang, Jianghua Wang, Xingwang Xie, Lai Wei

**Affiliations:** 1 Peking University People’s Hospital, Peking University Hepatology Institute, Beijing Key Laboratory of Hepatitis C and Immunotherapy for Liver Diseases, Beijing, China; 2 Liver Diseases Center, Beijing Ditan Hospital, Capital Medical University, Beijing, China; 3 Institute of Infectious Diseases, Beijing Ditan Hospital, Capital Medical University, Beijing Key Laboratory of Emerging infectious Diseases, Beijing, China; Saint Louis University, UNITED STATES

## Abstract

Currently, there is no effective vaccine to prevent hepatitis C virus (HCV) infection, partly due to our insufficient understanding of the virus glycoprotein immunology. Most neutralizing antibodies (nAbs) were identified using glycoprotein immunogens, such as recombinant E1E2, HCV pseudoparticles or cell culture derived HCV. However, the fact that in the HCV acute infection phase, only a small proportion of patients are self-resolved accompanied with the emergence of nAbs, indicates the limited immunogenicity of glycoprotein itself to induce effective antibodies against a highly evolved virus. Secondly, in previous reports, the immunogen sequence was mostly the genotype of the 1a H77 strain. Rarely, other genotypes/subtypes have been studied, although theoretically one genotype/subtype immunogen is able to induce cross-genotype neutralizing antibodies. To overcome these drawbacks and find potential novel neutralizing epitopes, 57 overlapping peptides encompassing the full-length glycoprotein E1E2 of subtype 1b were synthesized to immunize BALB/c mice, and the neutralizing reactive of the induced antisera against HCVpp genotypes 1–6 was determined. We defined a domain comprising amino acids (aa) 192–221, 232–251, 262–281 and 292–331 of E1, and 421–543, 564–583, 594–618 and 634–673 of E2, as the neutralizing regions of HCV glycoprotein. Peptides PUHI26 (aa 444–463) and PUHI45 (aa 604–618)-induced antisera displayed the most potent broad neutralizing reactive. Two monoclonal antibodies recognizing the PUHI26 and PUHI45 epitopes efficiently precluded genotype 2 viral (HCVcc JFH and J6 strains) infection, but they did not neutralize other genotypes. Our study mapped a neutralizing epitope region of HCV glycoprotein using a novel immunization strategy, and identified two monoclonal antibodies effective in preventing genotype 2 virus infection.

## Introduction

Hepatitis C virus (HCV) is one of the major causes of liver disease. An estimated 185 million people worldwide are infected with hepatitis C [1] and have a high risk of liver cirrhosis, hepatocellular cancer and death [2]. There is no prophylactic or therapeutic vaccine available for HCV, although rapid progress in hepatitis C treatment has been made due to the emergence of direct-acting antiviral (DAA) drugs. Once infected with HCV, most patients develop chronic hepatitis and only a small number of individuals clear the virus. Cellular immunity is thought to play a vital role in viral clearance [3–5]. Recently, accumulating evidence has highlighted the importance of humoral immunity in controlling infection [6,7]. Neutralizing antibodies (nAbs) were associated with the eradication of the virus both in the acute and chronic infection phases [7,8].

HCV glycoprotein, which mediates virus entry by interplay with host co-receptors, is the natural target of nAbs. Many nAbs with potent cross-genotype neutralizing reactive have been identified based on artificial glycoprotein immunogens, including recombinant E1E2, soluble E2, HCV pseudoparticles (HCVpp) and cell culture-derived HCV (HCVcc), mimicking the spare structure of the wild type virus glycoprotein [9–11]. Recently, the crystal structure of E2 was determined. The epitopes of these nAbs were mostly mapped to the “broadly neutralizing face”, mainly within the N terminal of E2 and approximately comprising amino acids (aa) 412–453 and 502–535 [12–14]. The E2-CD81 interaction region was also thought to be within this domain. The fact that only a few infected patients are resolved during the acute phase in the presence of nAbs implies that the epitopes recognized by the most potent and effective nAbs may be relatively weakly immunogenic and not reactive in most patients with hepatitis C. In the HCV E1E2 steric structure, the epitopes may be buried by adjacent conformation and not accessible for nAbs. On the contrary, variable regions of E2 are immunodominant [15], but they only raise strain-specific protective immunity, which is unable to neutralize highly evolved HCV [16]. Thus, the strategy of solely adopting a glycoprotein immunogen may miss some neutralizing epitopes outside the “broadly neutralizing face”. It is of interest to determine whether there are other novel neutralizing epitopes using a different immunization approach.

Another factor deserving attention is that, in previous studies, the glycoprotein sequence was based on the H77 strain, which represented the most prevalent genotype 1a worldwide. Other genotypes/subtypes were rarely studied, although theoretically one genotype/subtype immunogen was capable of inducing a cross-genotype nAbs [9], and the sera of chronic hepatitis C patients of one subtype were reported to have broadly neutralizing potential [17].

To address the issues mentioned above, we employed a different immunization strategy. First, we synthesized overlapping peptides encompassing the full-length glycoprotein E1E2 (not including the transmembrane domain of E2) instead of glycoprotein as the immunogen. Secondly, the immunogen sequence was mostly according to subtype 1b strain H77, which was prevalent globally and was the dominant subtype in China. Our study revealed that peptides of subtype 1b did induce nAbs, and the neutralizing epitopes of HCV glycoprotein were more broadly distributed than expected. Furthermore, we identified two monoclonal antibodies (mAbs), 2O18 and 2C21, recognizing epitopes aa 454–463 and aa 611–618 of E2, respectively, which efficiently blocked genotype 2 virus (HCVcc, JFH and J6 strains) infection *in vitro*.

Taken together, our study reveals the neutralizing domain of HCV glycoprotein from a new angle and also identifies two monoclonal antibodies that recognize novel glycoprotein epitopes blocking genotype 2 virus infection. These results facilitate future vaccine design and development.

## Materials and Methods

### Ethics Statements

All immunization procedures in BALB/c mice were conducted by Abmart Inc. (Shanghai, China; http://www.ab-mart.com) according to national guidelines (the Regulations for the Administration of Affairs Concerning Experimental Animals, China) and were approved by the Ethics Committee of Peking University People's Hospital.

### Peptide Synthesis

A peptide library consisting of 57 peptides (Tables 1 and 2) averaging 20 amino acid residues long and overlapping by 10 residues encompassing the complete sequence of HCV glycoprotein E1E2 (not including the transmembrane domain of E2, aa 718–746) of a subtype 1b “reference strain” was synthesized by Invitrogen Corp. (Shanghai, China). The “reference strain” was a consensus sequence generated by alignment of 43 chronic hepatitis C patient viral sequences belonging to subtype 1b (S1 File).

**Table 1:** Amino acid sequences of peptides PUHI 1–25 used to raise antibodies.

Peptide (PUHI)	aa Position	Sequence
1	192–211	YEVRNVSGVYHVTNDCSNSS
2	202–221	HVTNDCSNSSIVYEAADMIM
3	212–231	IVYEAADMIMHTPGCVPCVR
4	222–241	HTPGCVPCVRENNSSRCWVA
5	232–251	ENNSSRCWVALTPTLAARNA
6	242–261	LTPTLAARNASVPTTTIRRH
7	252–271	SVPTTTIRRHVDLLVGAAAF
8	262–281	VDLLVGAAAFCSAMYVGDLC
9	272–291	CSAMYVGDLCGSVFLVSQLF
10	282–301	GSVFLVSQLFTFSPRRHETV
11	292–311	TFSPRRHETVQDCNCSIYPG
12	302–321	QDCNCSIYPGHVSGHRMAWD
13	312–331	HVSGHRMAWDMMMNWSPTTA
14	322–341	MMMNWSPTTALVVSQLLRIP
15	332–353	LVVSQLLRIPQAVVDMVAGAHW
16	384–403	GTYVTGGAQAHTTRGFASLF
17	394–413	HTTRGFASLFTPGPSQKIQL
18	404–418	TPGPSQKIQLVNTNG
19	409–423	QKIQLVNTNGSWHIN
20	414–428	VNTNGSWHINRTALN
21	419–433	SWHINRTALNCNDSL
22	424–438	RTALNCNDSLNTGFL
23	429–443	CNDSLNTGFLAALFY
24	434–448	NTGFLAALFYTHKFN
25	439–453	AALFYTHKFNSSGCP

The amino acid positions are reported relative to the subtype 1a H77 strain.

**Table 2:** Amino acid sequences of peptides PUHI 26–57 used to raise antibodies.

Peptide (PUHI)	aa Position	Sequence
26	444–463	THKFNSSGCPERMASCRPID
27	454–473	ERMASCRPIDKFAQGWGPIT
28	464–483	KFAQGWGPITYAEPDSSDQR
29	474–493	YAEPDSSDQRPYCWHYAPRP
30	484–503	PYCWHYAPRPCGIVPASQVC
31	494–513	CGIVPASQVCGPVYCFTPSP
32	504–523	GPVYCFTPSPVVVGTTDRFG
33	514–528	VVVGTTDRFGVPTYN
34	519–533	TDRFGVPTYNWGENE
35	524–538	VPTYNWGENETDVLL
36	529–543	WGENETDVLLLNNTR
37	534–548	TDVLLLNNTRPPQGN
38	539–553	LNNTRPPQGNWFGCT
39	544–563	PPQGNWFGCTWMNSTGFTKT
40	554–573	WMNSTGFTKTCGGPPCNIGG
41	564–583	CGGPPCNIGGVGNNTLTCPT
42	574–593	VGNNTLTCPTDCFRKHPEAT
43	584–603	DCFRKHPEATYTKCGSGPWL
44	594–613	YTKCGSGPWLTPRCLVDYPY
45	604–618	TPRCLVDYPYRLWHY
46	609–623	VDYPYRLWHYPCTVN
47	614–628	RLWHYPCTVNFTIFK
48	619–633	PCTVNFTIFKVRMYV
49	624–643	FTIFKVRMYVGGVEHRLNAA
50	634–653	GGVEHRLNAACNWTRGERCD
51	644–663	CNWTRGERCDLEDRDRSELS
52	654–673	LEDRDRSELSPLLLSTTEWQ
53	664–683	PLLLSTTEWQILPCSFTTLP
54	674–693	ILPCSFTTLPALSTGLIHLH
55	684–703	ALSTGLIHLHQNIVDVQYLY
56	694–713	QNIVDVQYLYGVGSAVVSFA
57	704–717	GVGSAVVSFAIKWE

The amino acid positions are reported relative to the subtype 1a H77 strain.

### Animal Immunization and Antibody Generation

Fifty μg of peptide was used to inoculate BALB/c mice (n = 3 for each peptide) with complete adjuvant to elite polyclonal antibodies (antisera), and repeated at day 7, 14 and 21 with incomplete adjuvant. The antisera were collected 4 weeks post-immunization. The antibody concentration in the sera was validated by ELISA (>1:200). The production of the monoclonal antibody was conducted according to standard hybridoma technology. Ascites from inoculated BALB/c mice were collected at 4–6 weeks post-immunization, and monoclonal antibodies were purified from ascites using a protein A column. After the experiment, all mice were euthanized by CO_2_ asphyxiation.

### Cell Lines and Antibodies

HEK 293T (ATCC CRL-1573) and human Huh7.5 hepatoma cells were maintained and propagated as described previously [18]. Mouse anti-HCV NS3 was purchased from Abcam (Cat. 13830) and goat anti-mouse Alexa Fluor 488 was purchased from Invitrogen (Cat. A-11001). Normal mouse IgG was from Santa Cruz (SC-2025). The neutralizing antibodies CBH-5 and CBH-7 served as the positive controls [19].

### HCV Pseudoparticle (HCVpp) Production and Concentration

293T cells were co-transfected with expression plasmids encoding the HCV envelope glycoproteins, HIV gag/pol (pLP1), HIV rev (pLP2), and pcDNA3 encoding luciferase protein. HCV envelope expression plasmids included genotype 1a strain H77 (provided by F. L. Cosset, INSERM U758, Lyon, France), genotype 1b strain Con1 (provided by C. Rice, Rockefeller University, New York, NY), and genotypes 2a (clone UKN2A1.2), 3a (clone UKN3A1.28C), 4 (clone UKN4.21.16), 5 (UKN5.14.4) and 6 (UKN6.5.340) (provided by J. K. Ball, The University of Nottingham, United Kingdom). At 48 and 72 hours post-co-transfection, the virus-containing supernatants were harvested, filtered through 0.45 μm membranes, concentrated with a 100K Centrifugal Device (Pall, USA) and stored in aliquots at -80°C until use.

### Cell Culture Derived HCV (HCVcc) Production

Cell culture supernatant was collected from 10 μg full-length HCV RNA transfected Huh7.5 cells and was used to infect Huh7.5 cells grown in 100 mm dishes at a multiplicity of infection (MOI) of 0.01. The infected cells were passaged at 3-day intervals. At day 14 post-infection, viral supernatants were obtained and clarified by centrifugation and stored in aliquots at -80°C. The FFU of the HCVcc stock was measured using the end-point dilution method as described previously [11]. The HCVcc strains included J4 (1b), JFH (2a), J6 (2a), S52 (3a), ED43 (4a), SA13 (5a) and HK6a (6a) [20].

### Neutralization Assays

For HCVpp neutralization assays, 8×10^3^ Huh7.5 cells were seeded into 96-well plates one day before infection. Ten μL HCVpp stock was incubated with antiserum, monoclonal antibodies or normal mouse serum/IgG (control group) at various concentrations, plus 4 μg/ml polybrene at 37°C for 1 hour. The mixtures (100 μL in total) were then added to each well. After incubation at 37°C for 6 hours, the mixtures were replaced with complete culture medium and incubated for 72 hours. HCV infection was evaluated by measuring luciferase activity (Promega, Cat. E1501). The value of %Neutralization was calculated as (1- luciferase value of experimental group/luciferase value of control group) ×100%. The IC_50_ of the antibody (required to neutralize 50% of virus) was determined based on a neutralization curve generated from a series of 2-fold dilutions tested in triplicate.

For the HCVcc neutralization assays, 6×10^3^ Huh7.5 cells were seeded into 96-well plates one day before infection. A sample of 100 FFU HCVcc was incubated with monoclonal antibodies or normal mouse IgG (control group) at 37°C for 1 hour. The mixture was then incubated with Huh7.5 cells for 4 hours. Seventy-two hours post-infection, HCV infection was evaluated by counting HCV NS3-positive foci in an indirect immunofluorescence assay [21]. Each test was performed in triplicate. %Neutralization was calculated as (1- foci of experimental group/foci of control group) ×100%.

### Epitope Mapping

To map the precise epitopes of mAbs 2O18 and 2C21, three overlapping peptides covering aa 444–463 and aa 604–618, respectively, were synthesized. The plates were coated with 5 μg/ml of peptide and blocked with 4% PBST. The two mAbs were incubated and binding was detected in an ELISA format as described previously [22]. Irrelevant rabies virus peptide (VNLHDFRSDEIE) served as the negative control. Peptide PUHI26 (aa 444–463) and PUHI45 (aa 604–618) served as the positive controls.

### Alanine Replacement Mutagenesis

To identify residues crucial for mAbs binding, alanine mutagenesis of the epitope residues was analyzed in a GNL capture ELISA assay [23]. The OD value of antibody binding to epitope with replacing <50% of wild type residues was defined as positive. Each experiment was performed in triplicate.

### Data Analysis and Software

Statistical comparison of the neutralization between experimental and control groups was performed using the χ2 test (GraphPad Prism 5). The neutralizing entropy of 47 peptides inducing antisera against HCVpp was analyzed with Genesis software (http://genome.tugraz.at/genesisclient/genesisclient_description.shtml) [24].

## Results

### Neutralizing Epitope Domain of HCV Glycoprotein E1E2

A series of 57 overlapping peptides covering the full-length glycoprotein E1E2 (subtype 1b) was used as an immunogen to produce antibodies (Tables 1 and 2). The peptides were an average of 20-mers, with an overlap of 10 amino acids. Forty-seven of 57 peptides induced antisera containing high-titer polyclonal antibodies (>1:200 in ELISA). However, the remaining 10 peptides, including peptide PUHI 9 (aa 272–291), 10 (aa 282–301), 15 (aa 332–353), 18 (aa 404–418), 19 (aa 409–423), 24 (aa 434–448), 48 (aa 619–633), 55 (aa 684–703), 56 (aa 694–713) and 57 (aa 704–717), did not raise sufficient antibodies in BALB/c mice sera, although we repeated the immunization procedure 3 times (three mice for each peptide immunogen).

**Fig 1:**
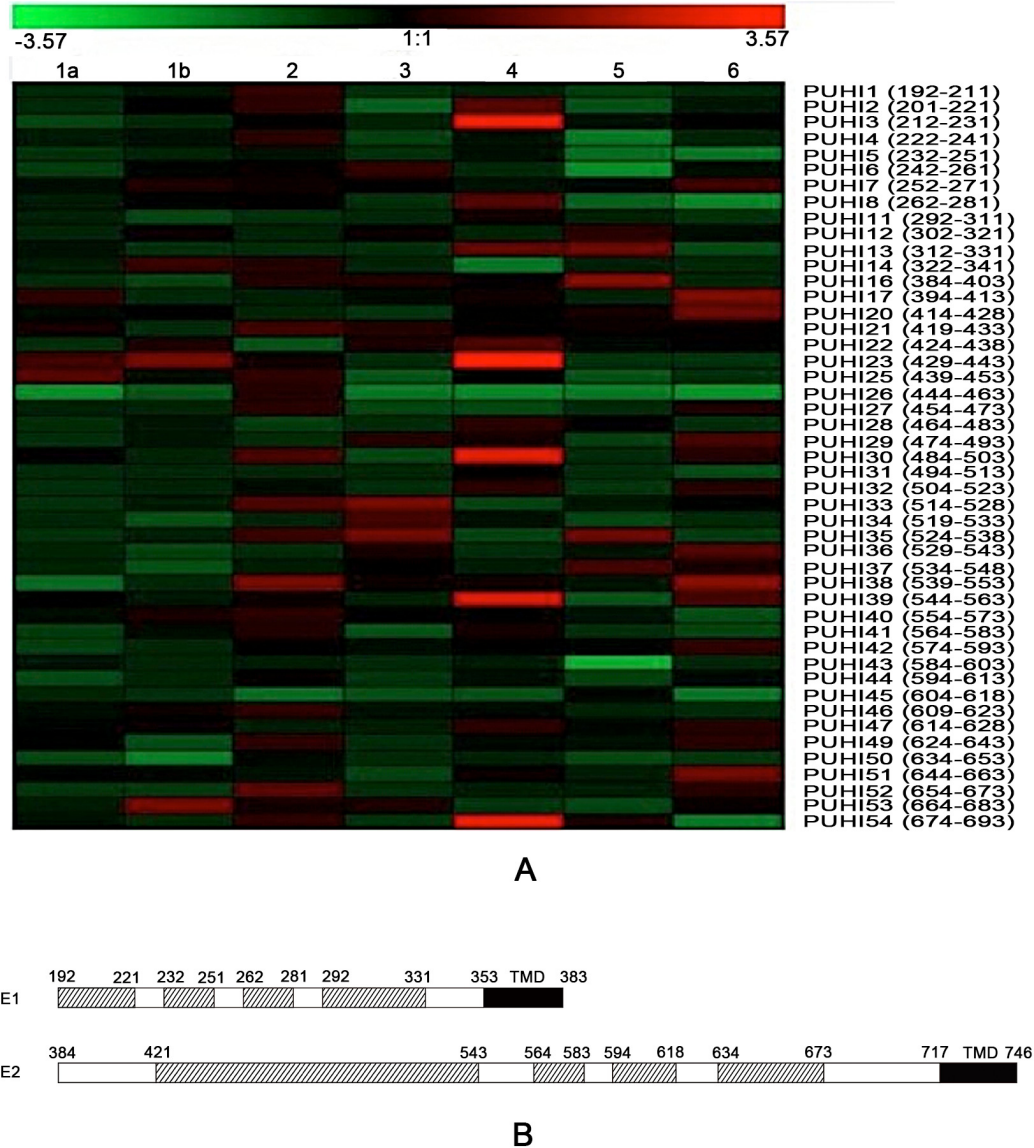
Neutralizing domain of HCV glycoprotein. (A) Neutralizing reactive of antisera (1:50 dilution) induced by overlapping peptides in the HCVpp neutralizing assay (genotypes/subtypes 1–6). PUHI indicates 47 peptides. The amino acid residue position of each peptide is shown in brackets. The relative neutralizing entropy of the antisera was analyzed with Genesis software and was represented with different kinds and degrees of colors. The green color indicates neutralizing reactive and the red color indicates promoting virus entry. “1:1” indicates that the antisera do not neutralize or promote virus infection. (B) Schematic diagram of the broadly neutralizing domain of the HCV glycoprotein in Tables 3 and 4. The broadly neutralizing domain is represented with slashes, including aa 192–221, 232–251, 262–281 and 292–331 of E1, and aa 421–543, 564–583, 594–618 and 634–673 of E2. The C terminal transmembrane domains of the glycoprotein (TMD) are shown in black.

To determine the neutralizing reactive of the antibodies induced by 47 peptides, the antisera were 50-fold diluted and tested in HCVpp neutralizing assays (genotypes 1–6). The relative neutralization of antisera was determined (Tables 3 and 4) and analyzed with Genesis software, as described previously [24]. The neutralizing values of the antisera were converted to different kinds and degrees of colors (Fig 1A). The green color indicated neutralizing reactive antisera, and the red color indicated antisera without neutralizing reactive or promoting virus entry.

**Table 3:** Average neutralization reactive of PUHI 1–22 induced antisera against genotypes/subtypes 1–6 HCVpp.

PUHI	% Neutralization against genotypes/subtypes 1–6 HCVpp						
	1a	1b	2	3	4	5	6
**1**	**30.6**	15.3	-50.6	**35.9**	22.2	**44.0**	28.9
**2**	**30.0**	-1.1	-43.8	**59.8**	-111.5	**54.0**	16.1
3	47.5	28.8	2.9	21.1	-745.1	13.3	-2.4
4	18.0	13.6	-37.9	44.7	12.3	70.1	24.4
**5**	**36.9**	22.5	12.4	**35.5**	11.3	**66.2**	**68.5**
6	43.2	5.1	-5.1	-54.6	28.7	75.3	9.9
7	10.6	-21.2	-8.3	2.0	13.4	6.6	-71.5
**8**	18.3	5.1	-4.3	**35.8**	-104.6	**60.5**	**73.3**
**11**	20.7	**48.2**	**35.0**	28.3	-9.6	21.5	**36.7**
12	23.9	-7.2	23.7	-7.5	21.8	-39.5	9.4
**13**	12.8	**35.1**	**32.3**	**34.0**	-133.9	-155.4	**49.1**
14	18.1	-58.1	-29.6	23.0	68.5	20.8	29.2
16	30.3	45.9	-26.4	-22.8	-5.6	-221.8	25.6
17	-42.9	29.2	20.3	9.0	-11.9	22.9	-202.6
20	6.9	-1.0	24.6	37.9	-4.1	-14.4	-156.7
21	-14.3	31.2	-65.9	-27.3	1.2	-6.1	-4.6
22	33.3	-21.2	54.8	-18.5	-99.8	6.9	-2.9

%Neutralization is calculated as (1-luciferase value of experimental group/luciferase value of control group) ×100%. Peptides inducing antisera with neutralization values ≥30% are in bold type.

**Table 4:** Average neutralization reactive of PUHI 23–54 induced antisera against genotypes/subtypes 1–6 HCVpp.

PUHI	% Neutralization against genotypes/subtypes 1–6 HCVpp						
	1a	1b	2	3	4	5	6
**23**	-69.0	-177.5	-10.4	**31.1**	-1091.6	**36.8**	**38.1**
**25**	-114.1	8.5	-47.5	**55.9**	3.1	**55.4**	**35.9**
**26**	**74.8**	**56.4**	-30.9	**69.5**	**73.5**	**70.5**	**76.1**
**27**	22.5	25.4	-39.7	**48.8**	**49.7**	**31.4**	-22.7
**28**	**34.1**	15.3	**42.6**	**29.7**	-39.6	2.6	**33.6**
**29**	**35.0**	15.3	**33.1**	-33.7	-14.6	**43.9**	-88.2
30	1.1	23.7	-83.0	47.7	-593.2	31.6	-30.5
**31**	**31.3**	27.9	26.4	28.3	-0.4	**33.3**	**50.8**
**32**	**34.4**	23.7	9.9	**33.6**	-36.2	**39.6**	-35.4
33	36.5	16.9	-99.7	-182.4	44.8	15.7	3.3
**34**	27.3	**53.5**	21.9	-86.2	12.2	**47.1**	**30.9**
**35**	**36.1**	20.3	-65.1	-169.1	**45.3**	-156.2	**42.3**
**36**	25.2	**46.5**	**31.7**	-17.2	**32.3**	14.0	-101.5
37	24.7	54.2	18.2	0.5	18.0	-78.1	-19.9
38	64.9	28.8	-181.9	-9.9	-10.6	13.8	-233.9
39	1.8	15.3	-6.2	19.6	-553.2	54.3	-84.3
40	9.8	-18.6	-39.4	1.9	3.4	12.8	53.5
**41**	**42.7**	8.8	-24.8	**54.0**	-16.6	**34.0**	**47.6**
42	44.6	28.3	0.3	10.1	2.6	80.3	-53.1
43	14.6	27.1	17.4	31.1	18.4	81.2	27.8
**44**	**49.8**	25.4	-5.1	**38.8**	11.0	**46.1**	7.8
**45**	25.0	**34.9**	**65.0**	**52.7**	**52.9**	5.9	**68.0**
46	9.8	-9.3	-46.6	12.2	8.3	22.3	32.1
47	4.8	-4.0	19.4	18.8	-41.2	6.3	-33.3
49	3.4	51.5	-43.4	33.4	7.0	12.7	-36.3
**50**	**47.2**	**70.1**	5.8	**30.8**	20.8	**48.2**	**37.4**
51	4.9	5.9	12.4	45.7	-7.5	19.4	-174.6
**52**	**37.8**	**40.2**	-121.3	**33.7**	18.8	19.0	-34.8
53	18.2	-184.3	-14.6	-23.7	39.7	47.4	-7.7
54	13.4	28.8	-45.8	36.2	-661.5	-20.6	69.0

%Neutralization is calculated as (1-luciferase value of experimental group/luciferase value of control group) ×100%. Peptides inducing antisera with neutralization values ≥30% are in bold type.

Our results revealed a huge difference in the neutralizing reactive of antisera induced by various peptides. Antisera induced by PUHI26 (aa 444–463) had the most potent neutralizing reactive against genotype 1a HCVpp (74.8%). The same antisera also showed different neutralizing activities against various genotypes of HCVpp; e.g., the neutralization of antisera induced by PUHI2 (aa 202–221) against genotypes 1a, 3, 5 and 6 HCVpp was 30.0%, 59.8%, 54.0% and 16.1%, respectively, but did not neutralize genotypes 1b, 2 and 4 HCVpp entry.

There is not a precise value of relative neutralization to define antisera as “neutralizing antisera”. In this study, we did not adopt rigorous standards (such as neutralization >50%) because during the neutralizing mAbs screening course, we found that the same peptide immunogen simultaneously induced neutralizing and non-neutralizing antibodies, and the latter somewhat promoted virus infection, which may offset the effect of neutralizing antibodies in antisera (S1 Fig). In our study, the difference between antisera with neutralizing reacitve ≥30% and the normal mice serum group (5.6%) was statistically significant (*P*< 0.01, χ2 test). In addition, the sera with neutralizing reactive ≥30% prevented virus entry in a dose-dependent manner (S2 Fig). Thus, we defined the antisera with relative neutralization ≥30% as “neutralizing antisera”.

Twenty-two peptides induced antisera that neutralized ≥30% against 3 genotypes/subtypes in the HCVpp neutralizing assay (Tables 3 and 4). Therefore, four regions of HCV E1 (aa 192–221, aa 232–251, aa 262–281 and aa 292–331) were defined as the broadly neutralizing domains. Four regions of HCV E2 (aa 421–543, aa 564–583, aa 594–618 and aa 634–673) were also defined as broadly neutralizing domains (Fig 1B).

**Fig 2:**
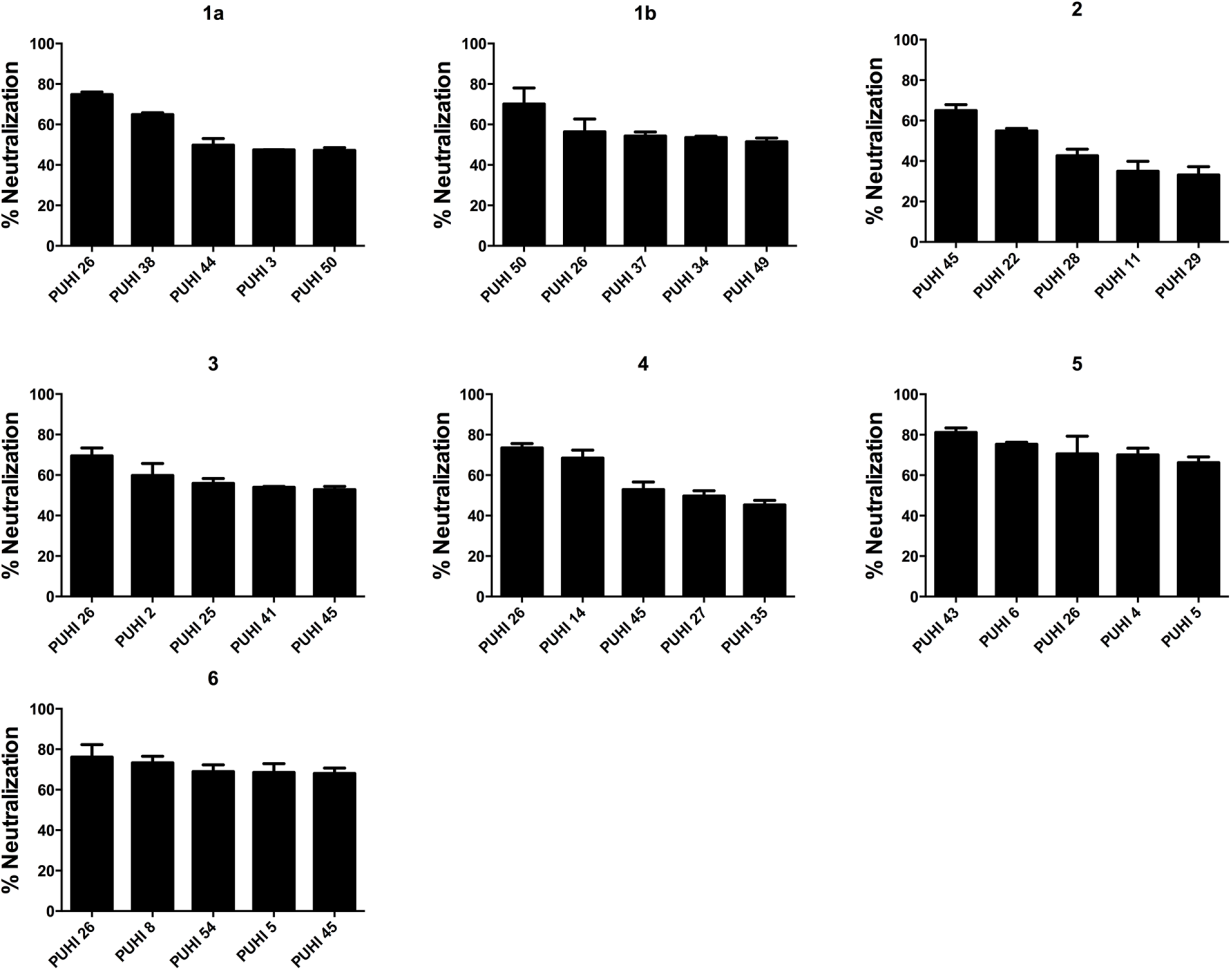
Antisera with the top five most potent neutralization against each genotype/subtype of HCVpp. The antisera were 1:50 diluted and assayed in the HCVpp neutralizing assay as described previously. The PUHI26-induced antiserum displayed the most potent neutralization against genotypes/subtypes 1a, 1b, 2, 3, 5, and 6 HCVpp, while the PUHI45-induced antiserum displayed the most potent neutralization against genotypes/subtypes 2, 3, and 6 HCVpp.

Fig 2 shows the peptides inducing antisera, with the top 5 most potent neutralizing reactive against genotypes/subtypes 1–6 HCVpp. Their epitopes were mostly within aa 421–543 of E2, followed by regions aa 594–618 and aa 564–583. Aa 421–543 represented the main domain, accounting for inducing the most potent neutralizing antisera against various genotypes of the virus. The neutralization of PUHI26 (aa 444–463)-induced antiserum against genotypes/subtypes 1a, 1b, 3, 4, 5 and 6 HCVpp was 74.8%, 56.4%, 69.5%, 73.5%, 70.5% and 76.1%, respectively. The neutralization of PUHI45 (aa 604–618)-induced antiserum against genotypes/subtypes 1a, 1b, 2, 3, 5, and 6 HCVpp at 25.0%, 34.9%, 65.0%, 52.7%, 52.9% and 68.0%, respectively. These two peptide immunogens induced the most potent broadly neutralizing antisera (Fig 2).

### Development of Monoclonal Antibodies Recognizing Neutralizing Epitopes

**Fig 3:**
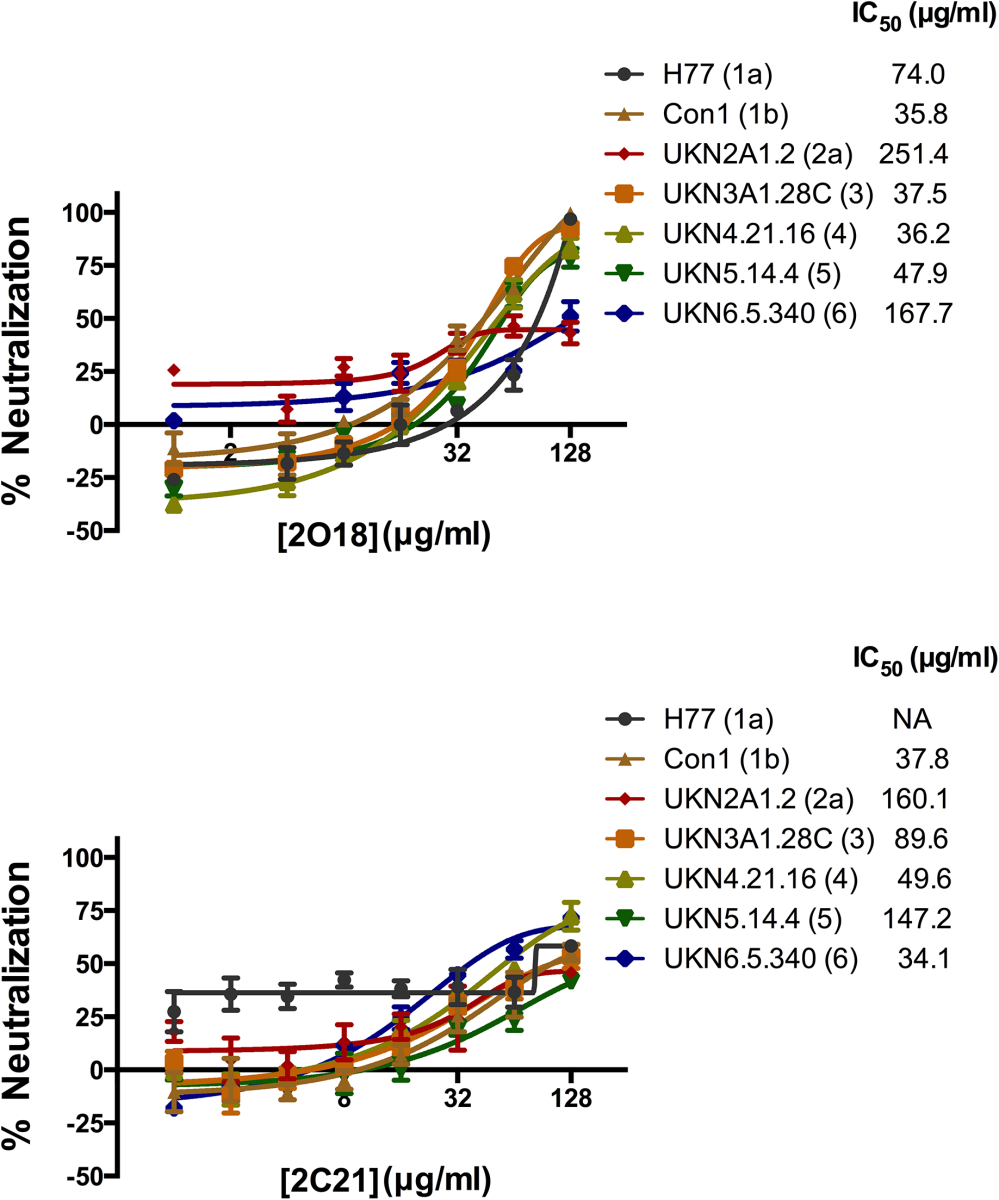
Dose-dependent neutralization of genotypes/subtypes 1–6 HCVpp with mAbs 2O18 and 2C21. 2O18 and 2C21 were 2-fold diluted, from 128 μg/ml to 1 μg/ml, mixed with HCVpp and incubated for 1 hour and then added to Huh7.5 cells. Seventy-two hours post-infection, viral infection was evaluated by determining luciferase activity. HCVpp without antibody served as the positive control. One-half inhibitory concentrations for virus neutralization values (IC_50_) against each HCVpp were calculated using GraphPad Prism software. NA, not applicable. The experiment was performed in triplicate and the error bars represented the standard error of the means (SEM).

Because the peptides PUHI26 (aa 444–463) and PUHI45 (aa 604–618) induced broadly neutralizing antisera, mAb isolates targeting the epitopes were produced using hybridoma techniques and tested in a HCVpp neutralizing assay (S1 Fig). Two mAbs, 2O18 and 2C21, recognizing aa 444–463 and aa 604–618, respectively, were identified as having neutralizing reactive (Fig 3). The neutralizing reactive of 2O18 against different genotypes of HCVpp varied, with the most potent neutralization against the con1 strain of genotype 1b (IC_50_ 35.8 μg/ml), and moderate neutralization against the genotype 6 strain (IC_50_ 167.7 μg/ml). Similarly, 2C21 displayed the most potent neutralizing reactive against genotype 6 virus (IC_50_ 34.1 μg/ml), but barely neutralized the genotype 1a H77 strain.

**Fig 4:**
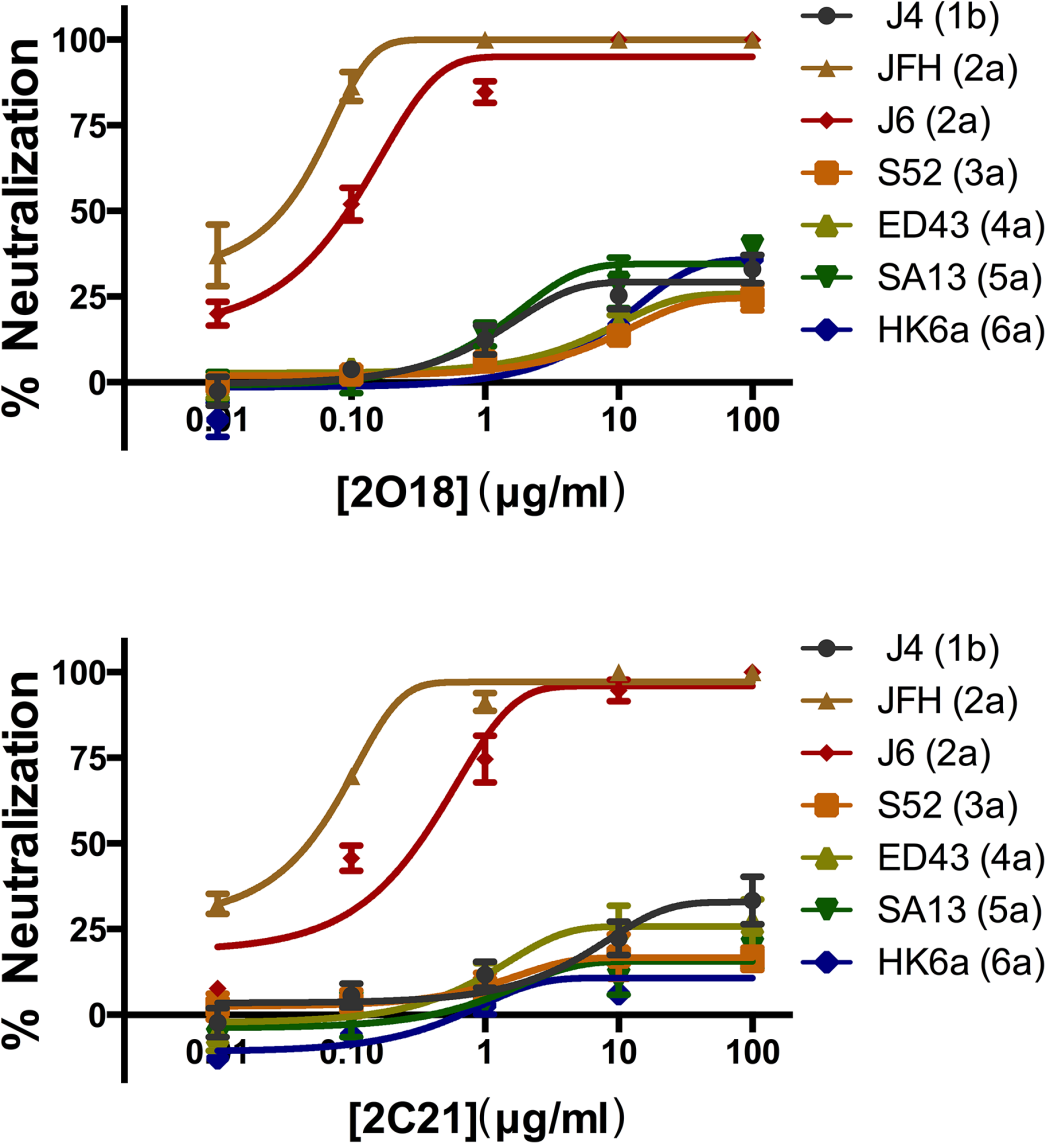
Dose-dependent neutralization of HCVcc genotype 2 strains JFH and J6 with mAbs 2O18 and 2C21. Antibodies were 10-fold diluted, from 100 μg /ml to 0.01 μg/ml, and assayed by HCVcc neutralization. All experiments were performed in triplicate and the error bars represented the standard error of the neutralization means (SEM).

To test 2O18 and 2C21 dose-dependent neutralizing reactive against various genotypes of HCVcc, they were serially 10-fold diluted from 100 μg/ml to 0.01 μg/ml and assayed in the HCVcc neutralizing assay. Both demonstrated potent neutralizing characteristics against genotype 2 JFH and J6 strains (Fig 4). However, they could not completely block other genotypes of HCVcc, including J4 (1b), S52 (3a), ED43 (4a), SA13 (5a) and HK6a (6a), even at 100 μg/ml.

### Epitope Mapping and Determination of Residues Crucial for Antibody Binding

**Fig 5:**
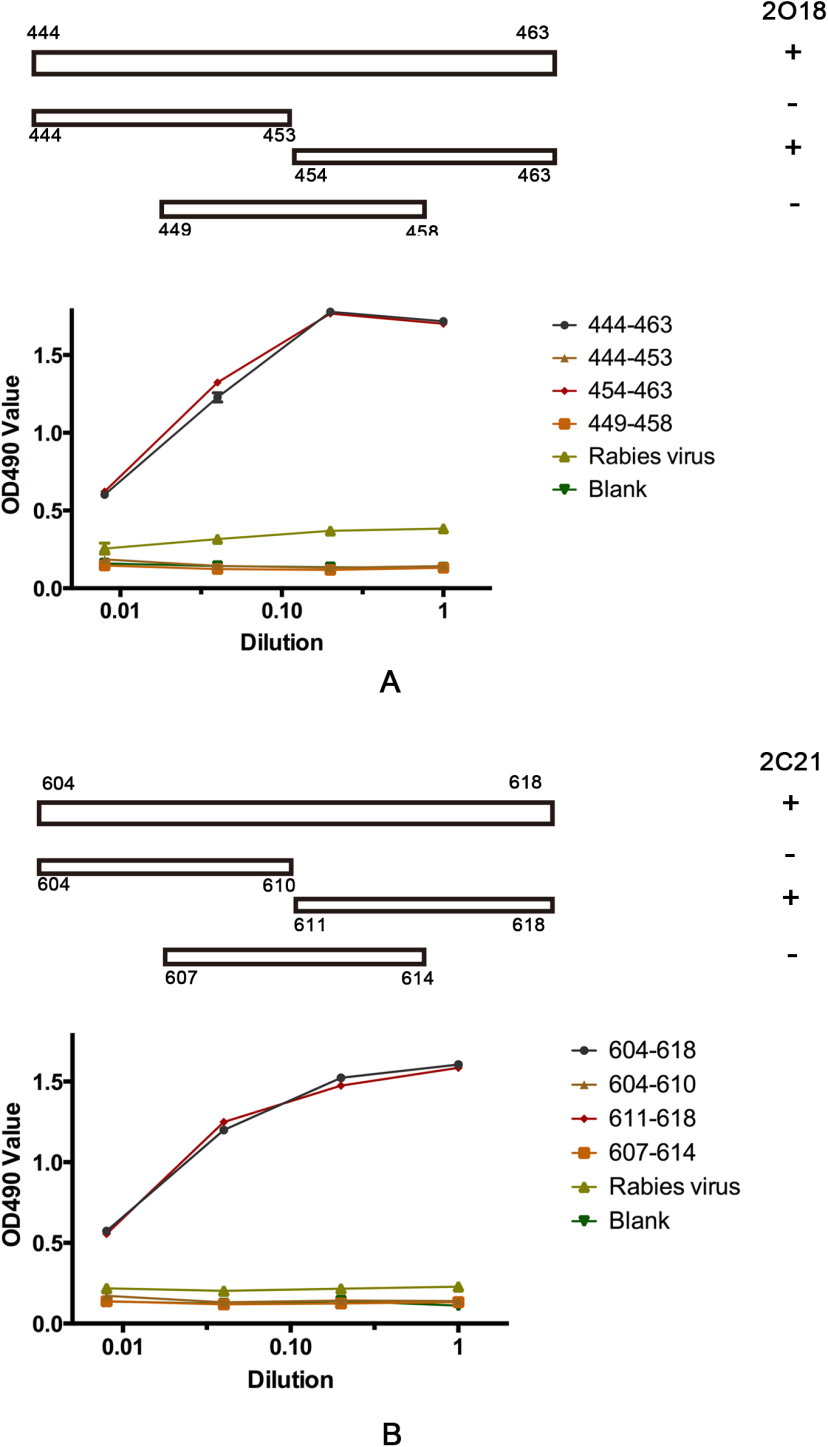
mAbs 2O18 and 2C21 epitope mapping. Three overlapping peptides, encompassing aa 444–463 and aa 604–618, were synthesized. The binding of mAbs to each peptide fragment was determined by ELISA. +, with binding activity.-, without binding activity. Peptides aa 444–463 and 604–618 served as the positive controls for 2O18 and 2C21, respectively, while the rabies virus peptide served as the negative control. Blank, without peptide.

To map the precise epitopes of 2O18 and 2C21, three overlapping peptides, encompassing aa 444–463 and aa 604–618, were synthesized and their relative binding to antibodies was determined in by ELISA (Fig 5). The results demonstrated that 2O18 and 2C21 recognized epitopes aa 454–463 and aa 611–618, respectively.

**Fig 6:**
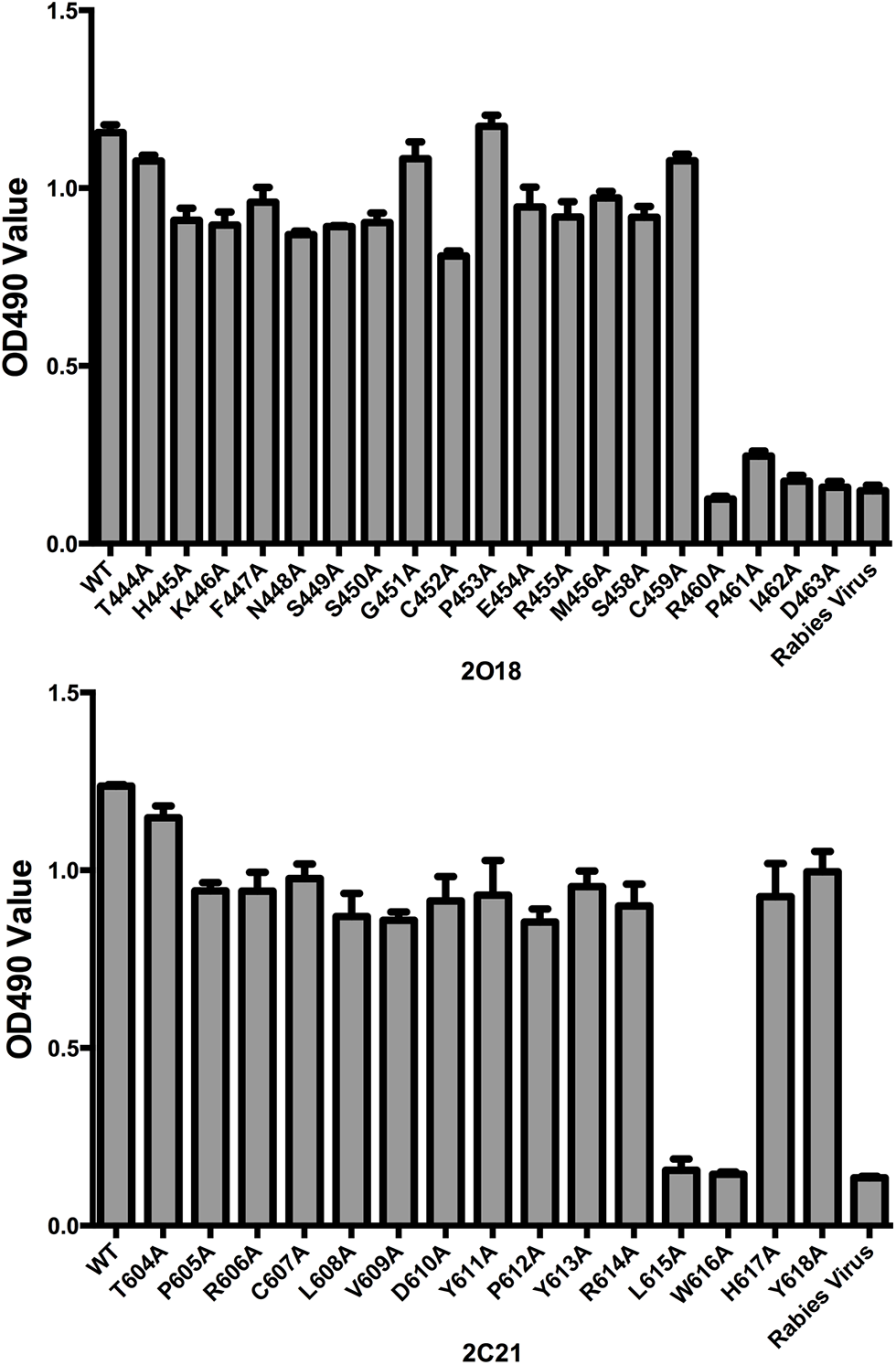
Alanine-replacement mutagenesis of the 2O18 and 2C21 epitopes. The amino acid residues mutated to alanine are shown. The relative binding of mAbs to each mutant was determined by ELISA and expressed as 490 nm OD value. WT (PUHI26 and PUHI45) and rabies virus peptide served as the positive and negative controls, respectively.

To determine the residues crucial for mAbs binding, we conducted alanine replacement mutagenesis (Fig 6). The results demonstrated that residues R460, P461, I462 and D463 were crucial for 2O18 binding, while L615 and W616 were responsible for 2C1 binding.

### Conservation Analysis of 2O18 and 2C21 Epitopes

**Fig 7:**
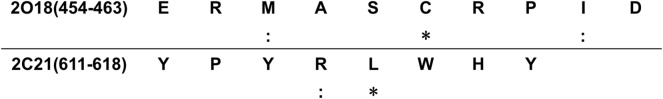
Conservation analysis of antibodies 2O18 and 2C21 epitopes. Five hundred and thirty-six HCV protein sequences from Table 5 were retrieved for alignment, and the conserved amino acid residues within 2O18 and 2C21 epitopes were marked. “:” indicates highly conserved residues, while “*” indicates absolutely conserved residues across 536 sequences.

To analyze the conservation of 2O18 and 2C21 epitopes, a total of 536 HCV sequences comprising genotypes/subtypes 1–6 were manually retrieved from the NCBI database (Table 5, S2 File). All the sequences were submitted to Clusta Omega (http://www.ebi.ac.uk/Tools/msa/clustalo/) for alignment, and the conserved epitope residues are marked in Fig 7. Residues M456, C459 and I462 of the 2O18 epitope were conserved, while R614 and L615 of the 2C21 epitope were conserved. Thus, the conserved residue crucial for 2O18 binding was I462, and L615 for 2C21.

**Table 5:** Sequences retrieved from the NCBI database. In total, 536 protein sequences comprising HCV genotypes/subtypes 1–6 were retrieved from the NCBI database for conservation analysis.

Genotypes/subtypes	Number
1a	85
1b	272
1c	2
2a	20
2b	82
2c	5
3a	37
3b	1
4	10
5	3
6	19
Total	536

## Discussion

Development of an effective vaccine against HCV, such as a neutralizing antibody, is an urgent need because of the inaccessibility and unaffordability of DAAs in many developing countries and regions in the near future. Neutralizing antibody plays an important role in controlling HCV infection and is associated with viral clearance [6,7]. Many nAbs were identified using glycoprotein immunogens, with the immunogen sequence according to the subtype 1a H77 strain. These epitopes were mostly mapped to a “neutralizing face”, mainly within the aa 412–535 region [12,25]. In this study, we adopted a different immunization strategy, using overlapping peptides instead of glycoproteins, and the 1b strain sequence instead of H77, to map the neutralizing domain of HCV glycoprotein. Our study revealed that the chief neutralizing domain of the glycoprotein was located at aa 421–543 of E2, which induced the most potent broadly neutralizing antisera, consistent with previously defined “neutralizing face”. The second neutralizing regions were aa 594–618 and aa 624–653 of E2. Others have also reported neutralizing epitopes at aa 611–616, aa 644–651 and aa 647–658 within the domains [26–28]. In addition, we mapped four neutralizing regions of E1, aa 192–221, aa 232–251, aa 262–281 and aa 292–331, although E2 was generally considered to be the immunodominant glycoprotein. Of note, there were some peptides within previously reported neutralizing epitopes, such as PUHI 19 (409–423) within epitope aa 412–423, that did not yield detectable antibody, although we repeated the immunization procedure. This may be due to the weak immunogenicity of the epitope. Indeed, epitope aa 412–423, recognized by nAb AP33, was detected in less than 2.5% of serum samples collected from acute and chronic hepatitis C patients [29]. The use of a particular carrier in combination with a peptide immunogen may improve the production of the antibody [30]. Of note, 2O18 and 2C21 antibodies appeared to promote infections of most tested HCVpp at low doses (<16μg/ml for 2O18, <8μg/ml for 2C21), the antibody Fc receptor was probably account for that [31].

Based on the identified neutralizing epitopes, we isolated two nAbs, 2O18 and 2C21. Both displayed cross-genotype neutralizing reactive in HCVpp neutralizing assays, and potently blocked HCVcc genotype 2 JFH and J6 strain infection. Epitope mapping revealed that some of the residues crucial for antibody binding were highly conserved, indicating that the interplay between antibodies and virus epitopes was relatively stable. Because genotype 2 is prevalent in the Asian-Pacific region and accounts for a large proportion of HCV infection, it is advisable to further test the efficacy of 2O18 and 2C21 in more HCVcc genotype 2 strains, and in vivo.

2O18 and 2C21 did not neutralize HCVcc of genotypes other than genotype 2, but displayed broad neutralizing reactive in HCVpp. The reason for this difference is elusive and needs further elucidation. The strains used in HCVpp and HCVcc were not exactly the same. Recently there were similar observations for other neutralizing antibodies [32].

In summary, our study reveals the neutralizing region of HCV glycoprotein by use of the peptide immunization strategy. We developed two monoclonal antibodies with potent neutralizing reactive against genotype 2 viruses. These findings have important implications for HCV vaccine design and development.

## Supporting Information

S1 Fig Neutralizing reactive of monoclonal antibody isolates against epitopes aa444-463 and aa604-618. 20 mice ascites monoclonal antibodies against epitope aa 444–463 (11365 and 11366 groups), and 30 monoclonal antibodies against epitope aa 604–618 (11367 and 11368 groups), were 1:50 diluted and tested in HCVpp neutralizing assay (genotype 1a, 1b and 2a). Some of the antibodies (clones 2–6 and 14 in 11365 group, and clones 7–14, CBH-5 in 11368 group) were not tested in genotype 2a HCVpp neutralizing asssay. Clone 2C21 in 11368 group was only tested in genotype 1b HCVpp neutralizing assay. Antibodies CBH-5 (13μg/ml) and CBH-7 (130μg/ml) were served as positive controls, respectively.
(TIFF)

S2 Fig Dose-dependent neutralization of HCVpp(genotype 1b). The sera were 2-fold diluted (started 1:50), and assayed by HCVpp neutralization. All experiments were performed in triplicate and the error bars represented the standard error of the neutralization means (SEM).
(TIFF)

S1 File Genotype 1b HCV sequences alignment (Bioedit 7.09). HCV genotype 1b reference sequence (aa192-717).
(ZIP)

S2 File HCV sequences conservation analysis (Bioedit 7.09). (ZIP)

## References

[1] Mohd Hanafiah K, Groeger J, Flaxman AD, Wiersma ST (2013) Global epidemiology of hepatitis C virus infection: new estimates of age-specific antibody to HCV seroprevalence. Hepatology 57: 1333–1342. 10.1002/hep.26141 23172780

[2] Lauer GM, Walker BD (2001) Hepatitis C virus infection. N Engl J Med 345: 41–52. 11439948 10.1056/NEJM200107053450107

[3] Claassen MA, Janssen HL, Boonstra A (2013) Role of T cell immunity in hepatitis C virus infections. Curr Opin Virol 3: 461–467. 10.1016/j.coviro.2013.05.006 23735335

[4] Rehermann B (2009) Hepatitis C virus versus innate and adaptive immune responses: a tale of coevolution and coexistence. J Clin Invest 119: 1745–1754. 10.1172/JCI39133 19587449 PMC2701885

[5] Walker CM (2010) Adaptive immunity to the hepatitis C virus. Adv Virus Res 78: 43–86. 10.1016/B978-0-12-385032-4.00002-1 21040831 PMC6171124

[6] Dowd KA, Netski DM, Wang XH, Cox AL, Ray SC (2009) Selection pressure from neutralizing antibodies drives sequence evolution during acute infection with hepatitis C virus. Gastroenterology 136: 2377–2386. 10.1053/j.gastro.2009.02.080 19303013 PMC2895772

[7] Osburn WO, Snider AE, Wells BL, Latanich R, Bailey JR, Thomas DL, et al (2014) Clearance of hepatitis C infection is associated with the early appearance of broad neutralizing antibody responses. Hepatology 59: 2140–2151. 10.1002/hep.27013 24425349 PMC4043926

[8] Ishii K, Rosa D, Watanabe Y, Katayama T, Harada H, Wyatt C, et al (1998) High titers of antibodies inhibiting the binding of envelope to human cells correlate with natural resolution of chronic hepatitis C. Hepatology 28: 1117–1120. 9755251 10.1002/hep.510280429

[9] Wong JA, Bhat R, Hockman D, Logan M, Chen C, Levin A, et al (2014) Recombinant hepatitis C virus envelope glycoprotein vaccine elicits antibodies targeting multiple epitopes on the envelope glycoproteins associated with broad cross-neutralization. J Virol 88: 14278–14288. 10.1128/JVI.01911-14 25275133 PMC4249152

[10] Deng Y, Guan J, Wen B, Zhu N, Chen H, Song J, et al (2013) Induction of broadly neutralising HCV antibodies in mice by integration-deficient lentiviral vector-based pseudotyped particles. PLoS One 8: e62684 10.1371/journal.pone.0062684 23626846 PMC3633868

[11] Akazawa D, Moriyama M, Yokokawa H, Omi N, Watanabe N, Date T, et al (2013) Neutralizing antibodies induced by cell culture-derived hepatitis C virus protect against infection in mice. Gastroenterology 145: 447–455 e441–444. 10.1053/j.gastro.2013.05.007 23673355

[12] Cashman SB, Marsden BD, Dustin LB (2014) The Humoral Immune Response to HCV: Understanding is Key to Vaccine Development. Front Immunol 5: 550 10.3389/fimmu.2014.00550 25426115 PMC4226226

[13] Khan AG, Whidby J, Miller MT, Scarborough H, Zatorski AV, Cygan A, et al (2014) Structure of the core ectodomain of the hepatitis C virus envelope glycoprotein 2. Nature 509: 381–384. 10.1038/nature13117 24553139 PMC4126800

[14] Liu R, Rao H, Wang J, Xie X, Jiang D, Pan X, et al (2013) Determination of the human antibody response to the neutralization epitopes encompassing amino acids 313–327 and 432–443 of hepatitis C virus E1E2 glycoproteins. PLoS One 8: e66872 10.1371/journal.pone.0066872 23826163 PMC3691243

[15] Puig M, Major ME, Mihalik K, Feinstone SM (2004) Immunization of chimpanzees with an envelope protein-based vaccine enhances specific humoral and cellular immune responses that delay hepatitis C virus infection. Vaccine 22: 991–1000. 15161076 10.1016/j.vaccine.2003.09.010

[16] Farci P, Shimoda A, Wong D, Cabezon T, De Gioannis D, Strazzera A, et al (1996) Prevention of hepatitis C virus infection in chimpanzees by hyperimmune serum against the hypervariable region 1 of the envelope 2 protein. Proc Natl Acad Sci U S A 93: 15394–15399. 8986822 10.1073/pnas.93.26.15394 PMC26415

[17] Pedersen J, Carlsen TH, Prentoe J, Ramirez S, Jensen TB, Forns X, et al (2013) Neutralization resistance of hepatitis C virus can be overcome by recombinant human monoclonal antibodies. Hepatology 58: 1587–1597. 10.1002/hep.26524 23729237 PMC4415732

[18] Keck Z, Wang W, Wang Y, Lau P, Carlsen TH, Prentoe J, et al (2013) Cooperativity in virus neutralization by human monoclonal antibodies to two adjacent regions located at the amino terminus of hepatitis C virus E2 glycoprotein. J Virol 87: 37–51. 10.1128/JVI.01941-12 23097455 PMC3536422

[19] Owsianka AM, Tarr AW, Keck ZY, Li TK, Witteveldt J, Adair R, et al (2008) Broadly neutralizing human monoclonal antibodies to the hepatitis C virus E2 glycoprotein. J Gen Virol 89: 653–659. 10.1099/vir.0.83386-0 18272755 PMC2885755

[20] Mathiesen CK, Jensen TB, Prentoe J, Krarup H, Nicosia A, Law M, et al (2014) Production and characterization of high-titer serum-free cell culture grown hepatitis C virus particles of genotype 1–6. Virology 458–459: 190–208. 10.1016/j.virol.2014.03.021 24928051 PMC4415741

[21] Meunier JC, Russell RS, Goossens V, Priem S, Walter H, Depla E, et al (2008) Isolation and characterization of broadly neutralizing human monoclonal antibodies to the e1 glycoprotein of hepatitis C virus. J Virol 82: 966–973. 17977972 10.1128/JVI.01872-07 PMC2224608

[22] Ndongo N, Berthillon P, Pradat P, Vieux C, Bordes I, Berby F, et al (2010) Association of anti-E1E2 antibodies with spontaneous recovery or sustained viral response to therapy in patients infected with hepatitis C virus. Hepatology 52: 1531–1542. 10.1002/hep.23862 20890942

[23] Ruwona TB, Giang E, Nieusma T, Law M (2014) Fine mapping of murine antibody responses to immunization with a novel soluble form of hepatitis C virus envelope glycoprotein complex. J Virol 88: 10459–10471. 10.1128/JVI.01584-14 24965471 PMC4178869

[24] Sturn A, Quackenbush J, Trajanoski Z (2002) Genesis: cluster analysis of microarray data. Bioinformatics 18: 207–208. 11836235 10.1093/bioinformatics/18.1.207

[25] Edwards VC, Tarr AW, Urbanowicz RA, Ball JK (2012) The role of neutralizing antibodies in hepatitis C virus infection. J Gen Virol 93: 1–19. 10.1099/vir.0.035956-0 22049091

[26] Clayton RF, Owsianka A, Aitken J, Graham S, Bhella D, Patel AH (2002) Analysis of antigenicity and topology of E2 glycoprotein present on recombinant hepatitis C virus-like particles. J Virol 76: 7672–7682. 12097581 10.1128/JVI.76.15.7672-7682.2002 PMC136371

[27] Owsianka A, Clayton RF, Loomis-Price LD, McKeating JA, Patel AH (2001) Functional analysis of hepatitis C virus E2 glycoproteins and virus-like particles reveals structural dissimilarities between different forms of E2. J Gen Virol 82: 1877–1883. 11457993 10.1099/0022-1317-82-8-1877

[28] Keck ZY, Xia J, Wang Y, Wang W, Krey T, Prentoe J, et al (2012) Human monoclonal antibodies to a novel cluster of conformational epitopes on HCV E2 with resistance to neutralization escape in a genotype 2a isolate. PLoS Pathog 8: e1002653 10.1371/journal.ppat.1002653 22511875 PMC3325216

[29] Tarr AW, Owsianka AM, Jayaraj D, Brown RJ, Hickling TP, Irving WL, et al (2007) Determination of the human antibody response to the epitope defined by the hepatitis C virus-neutralizing monoclonal antibody AP33. J Gen Virol 88: 2991–3001. 17947521 10.1099/vir.0.83065-0

[30] Torresi J, Fischer A, Grollo L, Zeng W, Drummer H, Jackson DC (2007) Induction of neutralizing antibody responses to hepatitis C virus with synthetic peptide constructs incorporating both antibody and T-helper epitopes. Immunol Cell Biol 85: 169–173. 17242693 10.1038/sj.icb.7100021

[31] Meyer K, Ait-Goughoulte M, Keck ZY, Foung S, Ray R (2008) Antibody-dependent enhancement of hepatitis C virus infection. J Virol 82: 2140–2149. 18094180 10.1128/JVI.01867-07 PMC2258956

[32] Carlsen TH, Pedersen J, Prentoe JC, Giang E, Keck ZY, Mikkelsen LS, et al (2014) Breadth of neutralization and synergy of clinically relevant human monoclonal antibodies against HCV genotypes 1a, 1b, 2a, 2b, 2c, and 3a. Hepatology 60: 1551–1562. 10.1002/hep.27298 25043937 PMC4415877

