# Supplementary figures and images for: Antibodies Targeting Novel Neutralizing Epitopes of Hepatitis C Virus Glycoprotein Preclude Genotype 2 Virus Infection
Source: PLoS One. 2015 Sep 25;10(9):e0138756. doi: 10.1371/journal.pone.0138756 (PMC4583415; doi:10.1371/journal.pone.0138756)

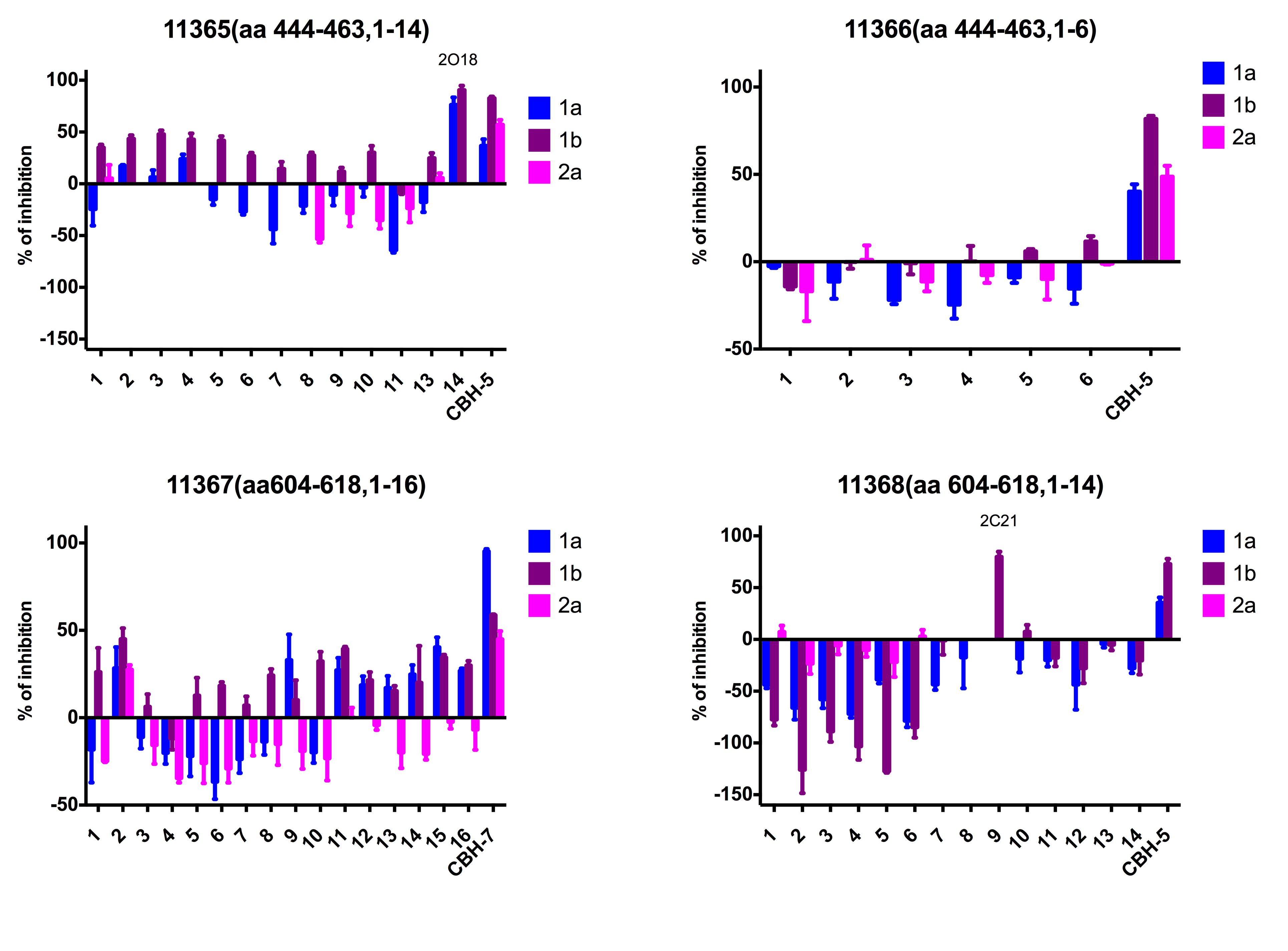

Supplement: S1 Fig — 20 mice ascites monoclonal antibodies against epitope aa 444–463 (11365 and 11366 groups), and 30 monoclonal antibodies against epitope aa 604–618 (11367 and 11368 groups), were 1:50 diluted and tested in HCVpp neutralizing assay (genotype 1a, 1b and 2a). Some of the antibodies (clones 2–6 and 14 in 11365 group, and clones 7–14, CBH-5 in 11368 group) were not tested in genotype 2a HCVpp neutralizing asssay. Clone 2C21 in 11368 group was only tested in genotype 1b HCVpp neutralizing assay. Antibodies CBH-5 (13μg/ml) and CBH-7 (130μg/ml) were served as positive controls, respectively. (TIFF) [file pone.0138756.s001.tiff]

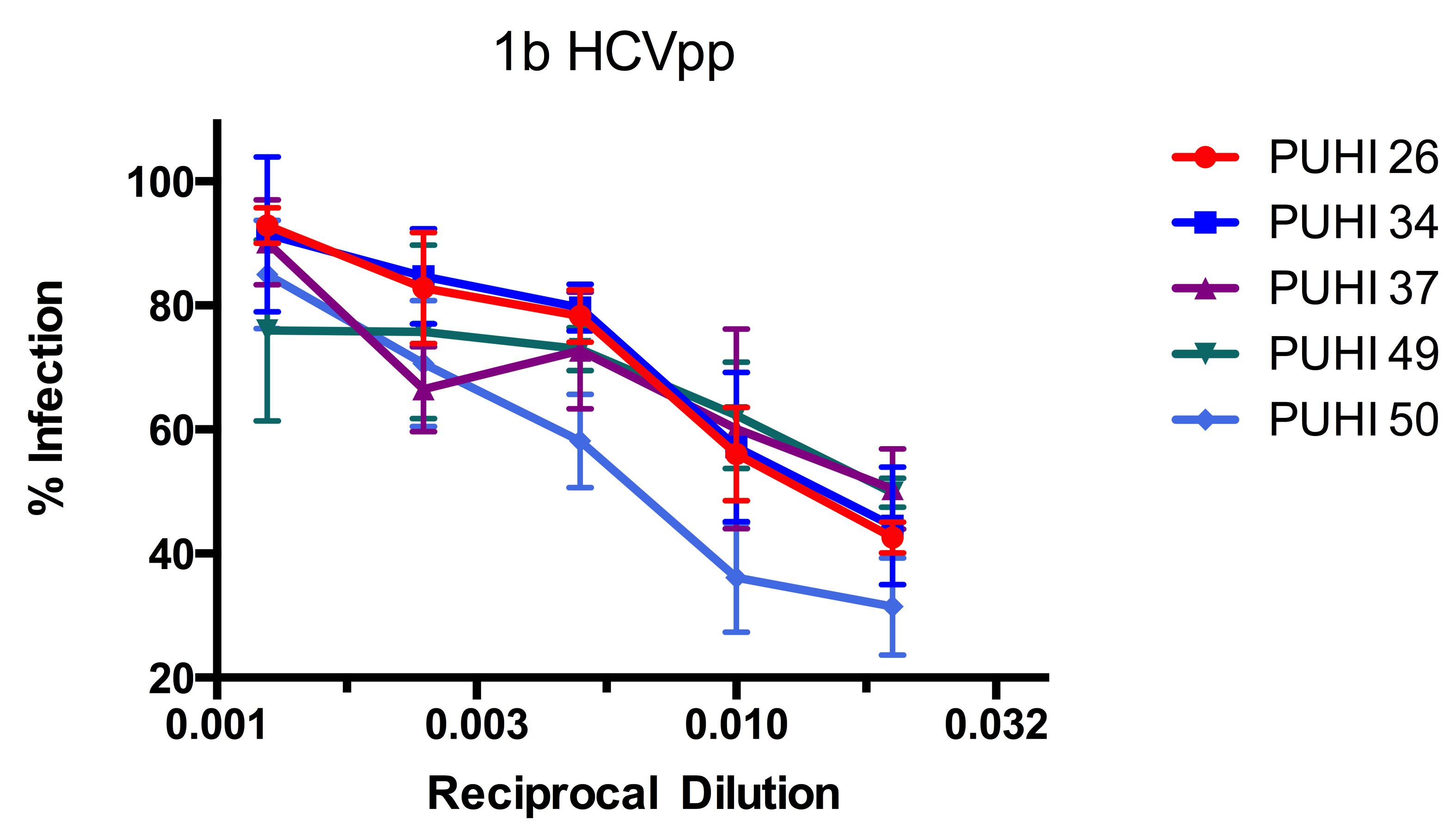

Supplement: S2 Fig — The sera were 2-fold diluted (started 1:50), and assayed by HCVpp neutralization. All experiments were performed in triplicate and the error bars represented the standard error of the neutralization means (SEM). (TIFF) [file pone.0138756.s002.tiff]
